# Acral Peeling Skin Syndrome: More than a Rare Genodermatosis Affecting Not Only the Skin

**DOI:** 10.3390/medicina62071368

**Published:** 2026-07-16

**Authors:** Aleksandra Kuźniak-Jodłowska, Jakub Szewczyk, Magdalena Jałowska, Karolina Grochowska, Grzegorz Nowaczyk, Aleksandra Dańczak-Pazdrowska

**Affiliations:** 1 Department of Dermatology, Poznan University of Medical Sciences, Przybyszewskiego 49, 60-356 Poznan, Poland; mjalowska@ump.edu.pl (M.J.); aleksandra.danczak-pazdrowska@ump.edu.pl (A.D.-P.); 2 Doctoral School, Poznan University of Medical Sciences, 60-812 Poznan, Poland; 3 NanoBioMedical Centre, Adam Mickiewicz University, Wszechnicy Piastowskiej 3, 61-614 Poznan, Poland; 4 Department of Medical Genetics, Poznan University of Medical Sciences, Rokietnicka 8 Street, 60-806 Poznan, Poland; kgrochowska654@gmail.com

**Keywords:** acral peeling skin syndrome, atomic force microscopy, genodermatosis

## Abstract

*Background and Objectives*: Acral peeling skin syndrome (APSS) is a rare autosomal recessive genodermatosis primarily affecting the skin. Although subtle hair abnormalities have been reported, data on hair morphology and age-related differences in APSS remain limited. *Materials and Methods*: Hair samples were collected from two pediatric patients with APSS aged 2 and 6 years and age-matched healthy controls. Five hair shafts from each participant were examined at two standardized distances from the root (5 mm and 15 mm). A total of ten atomic force microscopy (AFM) images were obtained for each hair shaft. Cuticle scale length, width, and deviation were analyzed using line profile measurements. Most analyses were performed on scan areas of 40 × 20 µm. *Results*: AFM revealed distinct nanoscale differences in hair cuticle morphology between children with APSS and healthy controls, including differences in cuticle scale step height, apparent cuticle scale length, and cuticle scale width. *Conclusions*: This exploratory study provides the first AFM characterization of hair shaft morphology in pediatric patients with APSS and provides a foundation for future research.

## 1. Introduction

Acral peeling skin syndrome (APSS) is a rare autosomal recessive genodermatosis characterized by superficial epidermal exfoliation and blistering, predominantly affecting the acral regions, including the volar and dorsal aspects of the hands and feet. The clinical manifestations are typically triggered by mechanical trauma, humidity, and increased temperature. The diagnosis may be challenging due to a nonspecific presentation, particularly in early childhood, when symptoms may overlap with other peeling or blistering disorders [1].

APSS is caused by mutations in the TGM5 gene encoding transglutaminase 5, an enzyme involved in the cross-linking of structural proteins during terminal keratinocyte differentiation and the formation of the cornified envelope. Impaired transglutaminase activity leads to reduced cohesion between corneocytes and increased fragility of the stratum corneum [2]. Disturbances in keratinization caused by TGM5 mutations may also affect other keratinized structures, including hair, as transglutaminase 5 plays a key role in protein cross-linking and terminal differentiation of keratinocytes, although hair abnormalities in APSS remain poorly characterized [3].

Experimental evidence suggests that specialized epithelial keratins expressed in the outer root sheath and companion layer are crucial for coordinated follicular differentiation and structural stabilization of the hair shaft, indicating that impaired keratinization, including defects related to TGM5, may result in subtle, age-dependent nanoscale alterations in hair structure [4]. In broader forms of peeling skin syndromes, hair fragility and easily detachable hair have been reported, and isolated observations suggest reduced hair density in patients with APSS [5].

These findings indicate that detailed hair analysis may provide additional insights into disease pathophysiology and structural abnormalities associated with impaired keratinization. Recent advances in imaging technologies have enabled high-resolution assessment of keratinized tissues, with atomic force microscopy (AFM) offering precise nanoscale characterization of hair structure [6,7]. Despite this potential, AFM studies of human hair have largely focused on healthy individuals, while data on rare genetic disorders and pediatric populations remain limited. In particular, age-related differences in nanoscale hair morphology have not been systematically investigated.

Therefore, the present study aimed to evaluate hair shaft morphology in children with acral peeling skin syndrome using AFM, with special emphasis on potential age-dependent structural differences. By comparing affected patients with healthy age-matched controls, we sought to determine whether nanoscale alterations in hair structure may reflect disturbances in keratinization and provide new insight into the pathophysiology and clinical variability of APSS.

## 2. Materials and Methods

### 2.1. Subjects

Two pediatric patients with a clinically and genetically confirmed diagnosis of acral peeling skin syndrome (APSS), aged 2 and 6 years, both female, were included in the study. The control group consisted of two healthy age- and sex-matched children. The inclusion criteria for the patient group were a clinically and genetically confirmed diagnosis of APSS. Healthy controls had no history of scalp or hair disorders. Exclusion criteria for all participants included hair dyeing or bleaching, the use of any medication or systemic disease known to affect hair structure or growth, and the use of dietary supplements intended to improve hair growth or quality. Five hairs were collected from each subject from comparable scalp regions. This exploratory study used minimal sample size estimation (Select Statistical Services, two-proportion calculator) assuming a large effect (α = 0.05; power = 80%), indicating that small groups may detect differences because APSS is an extremely rare disorder and morphological differences between patients with APSS and healthy controls are expected to be substantial, particularly in younger patients, although larger cohorts are needed to validate these findings. The study was approved by the Bioethics Committee at the Poznan University of Medical Sciences in Poznan (Resolution No. 930/23, dated 7 December 2023). Written informed consent was obtained from the legal guardians of all participants prior to enrolment.

### 2.2. Hair Preparation

Hair samples were collected non-invasively by cutting the hair shafts as close to the scalp as possible using sterile scissors. To ensure standardized sample collection and minimize the influence of external factors on hair surface morphology, participants were instructed not to wash their hair for at least 24 h before sampling. After the last hair wash, they were asked to avoid the use of hair care products (e.g., conditioners, masks, oils) and styling products (e.g., hair sprays, mousses, or gels). In addition, the use of heat-styling devices, including hair dryers, straighteners, curling irons, and crimpers, was prohibited for at least 24 h before sample collection.

### 2.3. Atomic Force Microscopy (AFM)

**Figure 1:**
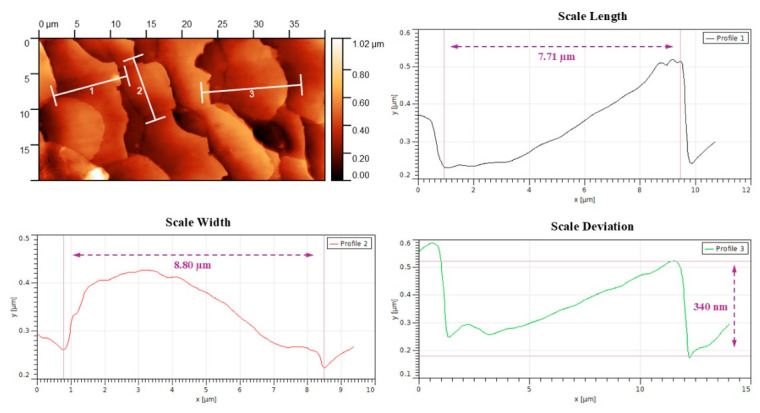
Parameters analyzed with AFM in terms of hair scale.

AFM was performed using a Bruker-Icon microscope under atmospheric conditions (in air) at room temperature. Imaging was performed in ScanAsyst mode, with Bruker SCANASYST-AIR (Silicon tip on Nitride Lever) tips. Tips were mounted on the cantilever, with a force constant (stiffness) of 0.4 N/m and a resonance frequency of 70 kHz. Each hair was imaged at a distance of 5 and 15 mm from the root. The scanning range was selected individually for each location to enable high-quality scanning without signal loss. Thus, the scanned areas ranged from 40 × 20 µm to 20 × 10 µm. The scanning velocity was 0.1 s/line. The resolution of the images was 512–1024 lines. At each of the 2 locations, at least 5 images were taken. Height profiles, image processing and all metric measurements (length, width, and scale deviation—Figure 1) were computer-processed using Gwyddion ver. 2.68 [8]. Scale length refers to the dimension parallel to the longitudinal axis of the hair. Due to the tile-like arrangement of the scales, only the portion of the scales available for examination was measured. Scale width refers to the transverse dimension of the longitudinal axis of the hair. To measure scale deviation, a line height profile of adjacent scales was made. Lines were run mostly perpendicular to the free end of each scale. From the resulting profile, the height difference in each step was measured.

### 2.4. Statistical Analysis

Statistical analysis was performed using IBM SPSS Statistics software, version 31.0.0. For each hair shaft, five AFM images were acquired at each distance from the hair root. Five independent measurements were performed on each image, and the mean value of these measurements was calculated. Each hair shaft at each distance from the hair root was treated as a single unit of analysis. The Shapiro–Wilk test was used to assess the distribution of data within the subgroups. As the data were not normally distributed, the exact Mann–Whitney U test was applied for subgroup comparisons. The results are presented as exact *p*-values and corresponding effect sizes (r). The statistical significance level was set at α = 0.05. The effect size (r) was calculated from the standardized Mann–Whitney U test statistic (Z) using the formula r = Z/√*N*, where *N* represents the total number of observations. For each comparison, *N* = 10, corresponding to five hair shafts from each of the two groups being compared. Effect sizes were interpreted according to commonly accepted thresholds, with values of approximately 0.1, 0.3, and 0.5 indicating small, moderate, and large effects, respectively.

## 3. Results

**Figure 2:**
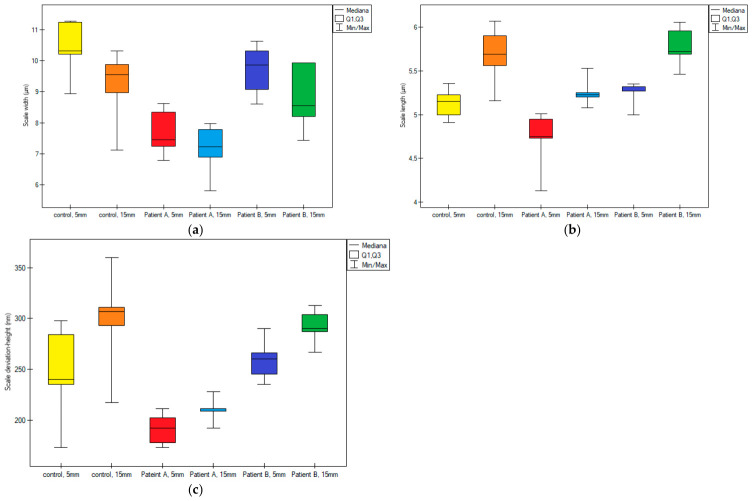
Box-and-whisker plots of cuticle morphometric parameters measured by AFM at 5 mm and 15 mm from the hair root in healthy controls and pediatric patients with APSS: scale width (**a**), scale length (**b**), and scale deviation (**c**).

A statistically significant difference in cuticle scale step height was demonstrated at a distance of 5 mm from the root between patient A and patient B (*p* = 0.01, r = 0.82; Figure 2). Although no statistically significant differences were demonstrated between patient A and the control group, the data analysis and the low *p*-value may indicate the presence of relevant differences that could not be confirmed due to the small sample size (*p* = 0.12). At a distance of 15 mm from the root, the cuticle scale step height differed significantly in patient A compared with both patient B (*p* = 0.01, r = 0.82) and the control group (*p* = 0.02, r = 0.70), demonstrating a large effect size. In both comparisons, patient A exhibited lower cuticle scale step height values than patient B and the healthy controls.

Similarly, for apparent cuticle scale length, statistically significant differences were observed between patient A and patient B, both for measurements taken at 5 mm from the root (*p* = 0.02, r = 0.72) and at 15 mm (*p* = 0.03, r = 0.76). In this case, the comparison between patient A and the control group also yielded results approaching statistical significance (*p* = 0.06 and *p* = 0.07 at 5 mm and 15 mm, respectively). Patient A consistently demonstrated shorter apparent cuticle scale lengths than both patient B and the control group. For cuticle scale width, significant differences were observed at 5 mm when comparing patient A with patient B (*p* = 0.02, r = 0.76) as well as patient A with the control group (*p* = 0.01, r = 0.83). At 15 mm, significant differences were found between patient A and patient B (*p* = 0.03, r = 0.69), while the comparison between patient A and the control group showed results approaching statistical significance (*p* = 0.06, r = 0.63). The cuticle scale width was consistently lower in patient A than in patient B and the healthy controls. The analysis did not reveal statistically significant differences between patient B and the control group for any of the evaluated parameters. Additionally, significant differences or trends toward statistical significance were observed between measurements obtained at 5 mm and 15 mm from the hair root, reflecting the physiological variability of hair shaft morphology along its length. The detailed morphometric measurements are presented in Table 1.

## 4. Discussion

Recent evidence suggests that APSS is associated with dysregulated epidermal proteolysis and impaired anchoring of endogenous protease inhibitors secondary to reduced transglutaminase 5 activity. These alterations lead to enhanced degradation of adhesion proteins, such as desmoglein-1, and compromise epidermal barrier integrity [9]. Experimental data further indicate that desmoglein-1 plays a critical role in maintaining hair anchorage within the hair follicle. In particular, overlapping expression of DSG1 and DSG3 in the companion layer is essential for structural cohesion between follicular compartments, and functional disruption of DSG1 has been shown to result in loss of anagen hair [10]. Therefore, increased proteolysis and degradation of DSG1 in APSS may not only affect epidermal adhesion but could also contribute to altered hair stability and keratinization, although this hypothesis requires further investigation.

In the literature, the variability in clinical presentation of APSS is emphasized, including marked differences in age of onset and disease severity, even among affected siblings. These observations highlight the significant phenotypic heterogeneity of this disorder [11]. Consistently, in our study, noticeable differences in clinical expression and nanoscale hair morphology were observed between patients despite the same diagnosis, supporting the concept of variable disease manifestation in APSS.

Previous studies have demonstrated upregulation of several epidermal differentiation markers, including keratin 1, keratin 10, involucrin, loricrin, and corneodesmosin, in APSS skin, suggesting the presence of compensatory mechanisms aimed at stabilizing the epidermal barrier. These adaptive processes may partly explain the clinical variability of the disease and the tendency toward milder manifestations in some older patients [12].

Our findings suggest age-related differences in hair cuticle morphology in children with APSS, with nanoscale features in the older patient being more similar to those observed in healthy controls. These observations are consistent with clinical reports suggesting that the severity of APSS may decrease with age [13]. However, this observation should be interpreted with caution because of the exploratory nature of the study and the limited number of participants. To our knowledge, this is the first study using AFM to characterize hair shaft morphology in pediatric patients with APSS. Previous AFM studies have mainly focused on healthy hair or common dermatological conditions, while data on rare genodermatoses remain limited [14]. Our results suggest that disturbances in keratinization associated with TGM5 mutations may also affect hair structure. Transglutaminase 5 plays an important role in epidermal differentiation and protein cross-linking in keratinized tissues, which may explain the observed structural abnormalities [15].

Currently, the diagnosis of APSS is based primarily on the characteristic clinical presentation and is confirmed by molecular genetic testing, most commonly through the identification of pathogenic variants in the TGM5 gene [2,11]. Therefore, AFM cannot replace the current diagnostic approach. However, it may serve as a complementary research tool for the detailed characterization of hair shaft morphology and may contribute to a better understanding of the pathophysiology of APSS. Although the present findings are encouraging, they do not support the incorporation of AFM into the diagnostic criteria for APSS. Larger studies comparing APSS with other inherited hair disorders are required before any diagnostic application can be considered.

The assessment of hair morphology represents an important yet underexplored aspect of genodermatoses. While characteristic hair shaft abnormalities have been described in Netherton syndrome, monilethrix, trichothiodystrophy, pili torti, and ectodermal dysplasias, most investigations have relied on conventional microscopy techniques. As a result, detailed quantitative analysis at the nanoscale has not been systematically performed in these inherited disorders [16,17]. Whether the nanoscale alterations observed in APSS are disease-specific or represent common features of inherited disorders affecting keratinization remains to be established.

In our study, we performed quantitative morphometric analysis of hair in patients with APSS, including cuticle scale length, cuticle scale width, and cuticle scale step height, with cuticle scale deviation being quantified as cuticle scale step height in accordance with previous AFM studies [6,18].

These findings differ in part from a report in patients with lichen planopilaris (LPP), in which an overall increasing trend in scale step height, apparent scale length, and scale width along the hair shaft was described. However, detailed analysis of their results reveals that local fluctuations in scale width were also present. In particular, decreases in scale width were observed in the LPP group between 2 and 3 cm and between 4.5 and 6.5 cm, and in the control group between 0.5 and 1 cm as well as between 4.5 and 5.5 cm from the root [18].

**Figure 3:**
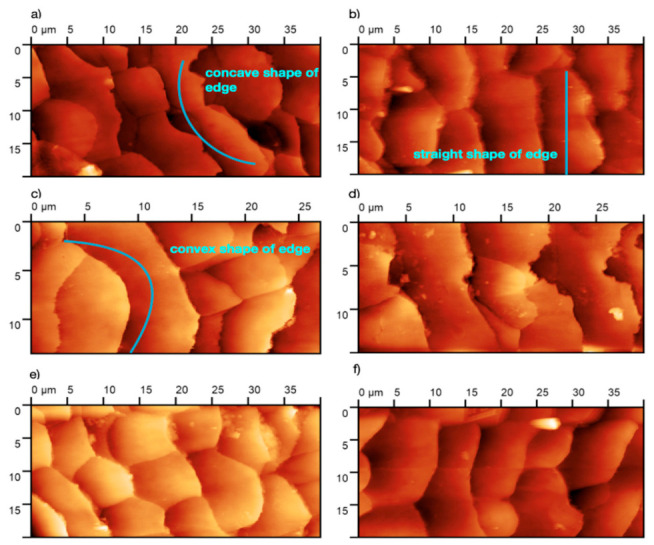
Representative AFM images of hair cuticle edges at 5 mm (**a**,**c**,**e**) and 15 mm (**b**,**d**,**f**) from the hair root; panels (**a**,**b**) correspond to Patient A, (**c**,**d**) to Patient B, and (**e**,**f**) to the control group.

Moreover, in their study on virgin Caucasian hair, a reduction in scale width was also noted between 0.5 cm and 1.5 cm from the root. Specifically, scale width decreased from 17,234.0 nm at 0.5 cm to 17,177.0 nm at 1.5 cm, despite the overall increasing trend observed across the full hair length. This observation is consistent with our findings, where a decrease in scale width was also detected in the proximal hair segments [6]. Taken together, these findings indicate that cuticle morphometric parameters do not change linearly along the hair shaft but rather demonstrate segmental variability. Therefore, the reduction in scale width observed in our APSS cohort between 5 mm and 15 mm from the root may reflect physiological or structural heterogeneity rather than a strictly disease-specific pattern. In addition to these morphometric differences, progressive deterioration of cuticle edge morphology was also observed with increasing distance from the hair root. In proximal segments, the free margins of the scales appeared smooth, rounded, and convex. In more distal regions, the edges became irregular, frayed, and partially disrupted, with a gradual transition toward a concave, C-shaped configuration. These changes reflected progressive structural wear and are presented in Figure 3 [6]. At the same time, the presence of statistically significant differences between APSS patients and controls suggests that disease-related alterations in keratinization may influence the dynamics of cuticle remodelling at the nanoscale.

The main limitation of this study is the small sample size, reflecting the exceptional rarity of APSS. Consequently, the present exploratory study was not powered to draw robust conclusions regarding age-related differences or inter-patient variability. Therefore, the reported *p*-values and effect sizes should be interpreted with caution and regarded as hypothesis-generating rather than confirmatory. In addition, AFM is a relatively expensive and time-consuming technique, and the acquisition, processing, and interpretation of AFM images require specialized equipment and considerable expertise. These factors currently limit the widespread implementation of AFM in routine clinical practice. Future studies involving larger pediatric cohorts, direct comparisons between healthy children and adults, and other inherited hair disorders are needed to determine whether the observed nanoscale alterations are specific to APSS or represent age-dependent differences, physiological structural variability, or early markers of genodermatosis-related hair involvement.

## 5. Conclusions

In conclusion, this study highlights the potential of AFM as a valuable and non-invasive technique for detailed morphological assessment in APSS, providing preliminary insights that may contribute to a better understanding of hair abnormalities in rare keratinization disorders and paving the way for more comprehensive future investigations. Further studies in larger groups are necessary to confirm these findings. Future research should include comparisons between pediatric and adult patients, as well as between healthy children and healthy adults, to better understand age-related changes in keratinization and hair structure. In addition, combining AFM with nanomechanical and molecular analyses may improve the understanding of disease mechanisms in APSS. Specifically, methods such as AFM-IR or quantitative mechanical property mapping may in the future find application for similar research and examination procedures.

Overall, the present study provides a foundation for future research and highlights the potential role of AFM as a non-invasive tool for the investigation of rare keratinization disorders.

## Figures and Tables

**Table 1:** Morphometric analysis of hair cuticle parameters in APSS patients and controls.

Patient	Distance from the Root	Scale Step Height (µm)	Apparent Scale Length (µm)	Scale Width (µm)
Patient A	5 mm	192 (178–202)	4.75 (4.73–4.95)	7.45 (7.24–8.35)
	vs. control 5 mm	*p* = 0.12, r = 0.53	*p* = 0.06, r = 0.63	*p* = 0.01 *, r = 0.83
	vs. patient B 5 mm	*p* = 0.01 *, r = 0.82	*p* = 0.02 *, r = 0.72	*p* = 0.02 *, r = 0.76
	15 mm	209 (209–211)	5.23 (5.2–5.25)	7.23 (6.89–7.79)
	vs. control 15 mm	*p* = 0.02 *, r = 0.70	*p* = 0.07, r = 0.52	*p* = 0.06, r = 0.63
	vs. patient B 15 mm	*p* = 0.01 *, r = 0.82	*p* = 0.03 *, r = 0.76	*p* = 0.03 *, r = 0.69
	5 mm vs. 15 mm	*p* = 0.16, r = 0.46	*p* = 0.01 *, r = 0.83	*p* = 0.42, r = 0.33
Patient B	5 mm	260 (245–266)	5.27 (5.27–5.32)	9.87 (9.07–10.32)
	vs. control 5 mm	*p* = 0.76, r = 0.13	*p* = 0.36, r = 0.33	*p* = 0.36, r = 0.32
	15 mm	290 (287–304)	5.72 (5.69–5.96)	8.56 (8.21–9.93)
	vs. control 15 mm	*p* = 0.55, r = 0.23	*p* = 0.76, r = 0.13	*p* = 0.84, r = 0.09
	5 mm vs. 15 mm	*p* = 0.04 *, r = 0.66	*p* = 0.01 *, r = 0.83	*p* = 0.22, r = 0.43
Control	5 mm	240 (235–284)	5.15 (5.00–5.23)	10.32 (10.21–11.24)
	15 mm	307 (293–311)	5.69 (5.56–5.90)	9.55 (8.98–9.89)
	5 mm vs. 15 mm	*p* = 0.15, r = 0.60	*p* = 0.03 *, r = 0.69	*p* = 0.18, r = 0.47

Results are presented as median and quartiles. Abbreviation: APSS-Acral Peeling Skin Syndrome, Patient A (6 years); Patient B (2 years), *p*-statistical significance, r-effect size, each value represents the median and interquartile range (IQR) calculated from five hair shafts analyzed for each subject at each distance from the hair root. Each hair shaft was treated as a single unit of analysis. * Indicates statistical significance at *p* < 0.05.

## Data Availability

The data are not publicly available due to privacy.

## Abbreviations

The following abbreviations are used in this manuscript:

AFM	Atomic Force Microscopy
APSS	Acral peeling skin syndrome
DSG1	Desmoglein 1
DSG3	Desmoglein 3
